# BiOI-SnO_2_ Heterojunction Design to Boost Visible-Light-Driven Photocatalytic NO Purification

**DOI:** 10.3390/ijerph20054009

**Published:** 2023-02-23

**Authors:** Han Chen, Yutao Hu, Zanyun Ying, Yinfeng Xia, Jiexu Ye, Jingkai Zhao, Shihan Zhang

**Affiliations:** 1 Key Laboratory for Technology in Rural Water Management of Zhejiang Province, Zhejiang University of Water Resources and Electric Power, Hangzhou 310018, China; 2 Key Laboratory of Microbial Technology for Industrial Pollution Control of Zhejiang Province, College of Environment, Zhejiang University of Technology, Hangzhou 310014, China; 3 College of Science & Technology, Ningbo University, Ningbo 315212, China

**Keywords:** NO removal, photocatalysis, BiOI/SnO_2_, heterojunction, enhanced mechanism

## Abstract

The efficient, stable, and selective photocatalytic conversion of nitric oxide (NO) into harmless products such as nitrate (NO_3_^−^) is greatly desired but remains an enormous challenge. In this work, a series of BiOI/SnO_2_ heterojunctions (denoted as *X*%B-S, where *X*% is the mass portion of BiOI compared with the mass of SnO_2_) were synthesized for the efficient transformation of NO into harmless NO_3_^−^. The best performance was achieved by the 30%B-S catalyst, whose NO removal efficiency was 96.3% and 47.2% higher than that of 15%B-S and 75%B-S, respectively. Moreover, 30%B-S also exhibited good stability and recyclability. This enhanced performance was mainly caused by the heterojunction structure, which facilitated charge transport and electron-hole separation. Under visible light irradiation, the electrons gathered in SnO_2_ transformed O_2_ to ·O_2_^−^ and ·OH, while the holes generated in BiOI oxidized H_2_O to produce ·OH. The abundantly generated ·OH, ·O_2_^−^, and ^1^O_2_ species effectively converted NO to NO^−^ and NO_2_^−^, thus promoting the oxidation of NO to NO_3_^−^. Overall, the heterojunction formation between p-type BiOI and n-type SnO_2_ significantly reduced the recombination of photo-induced electron-hole pairs and promoted the photocatalytic activity. This work reveals the critical role of heterojunctions during photocatalytic degradation and provides some insight into NO removal.

## 1. Introduction

The majority of the world’s population lives in areas where the air quality level exceeds the World Health Organization (WHO) criterion [1,2]. Although air pollution is not visible, it causes severe damage to human health and the environment [3]. Nitrogen oxides (NO*_x_*) are typical air pollutants that are not only harmful at concentrations as low as 21 ppb (40 μg m^−3^) [4] and are also deemed responsible for multiple atmospheric environmental issues, including photochemical smog, acid rain, and haze [5,6,7]. The efficient reduction of ambient NO*_x_* concentrations is therefore urgent in developing countries. In its 14th Five-Year Plan, the Chinese government set a target to lower NO*_x_* concentrations by over 10% in 2025 compared with 2020. About 90–95% of total NO*_x_* emissions consist of nitric oxide (NO). Therefore, developing effective NO elimination technologies would be of great significance [8]. 

The removal of NO via photocatalytic technology shows good promise because photocatalytic methods are environmentally friendly and cost-effective compared with conventional NO removal approaches such as selective catalytic reduction (SCR). Currently, the photocatalytic conversion of NO to harmless nitrate (NO_3_^−^) is under investigation, but this strategy is hampered by the low conversion efficiency and toxic/undesired by-product formation [9,10]. NO is inevitably transformed into nitrogen dioxide (NO_2_), which is more harmful than NO. Therefore, promoting the reaction selectivity to enhance the generation of NO_3_^−^ as the main product is an important problem that still remains to be solved for the successful photocatalytic removal of NO [11,12]. 

Metal oxide semiconductors such as TiO_2_, ZnO, and SnO_2_ have received extensive attention due to their simple preparation, intensive sources, low cost, and high stability [13]. However, the application of single photocatalysts is restricted by low visible light absorption and detrimental electron-hole recombination [14,15]. The combination of materials is a valuable strategy for improving the utilization of sunlight and the separation of photogenerated charge carriers, thus boosting the photocatalytic activities [16]. It is found that the TiO_2_/Nb_2_O_5_ heterostructure can improve the NO photodegradation by 7.0-folds and 3.8-folds higher removal efficiency, compared with pure Nb_2_O_5_ and TiO_2_, respectively [17]. Moreover, the ZnO/rGO composite is confirmed to possess remarkable performance in gaseous acetaldehyde degradation, which is 1.6 times superior to that of pure ZnO [18]. SnO_2_ is regarded as one of the best n-type direct band gap semiconductor photocatalysts. It possesses good stability, outstanding optical properties, and excellent electronic properties [19]. The proportion of visible light (50%) in the solar spectrum is far greater than that of ultraviolet light (4%). However, SnO_2_ can only be excited by ultraviolet light because of its wide band gap (3.5–3.7 eV) [20,21]. Hence, broadening the spectrum of light able to be absorbed by SnO_2_ would help realize its practical application. Among the various modification methods, photosensitization presents an exciting strategy for efficiently promoting visible light photocatalysis. In this method, a photosensitizer that matches the band structure of the semiconductor with a wide band gap is selected [22,23]. Bismuth oxyhalides BiOX (X = Cl, Br, and I) can act as photosensitizers with narrow band gaps and superior characteristics under visible light [24]. Therefore, the light absorption spectrum of SnO_2_ should be broadened into the visible light range by designing heterostructured photocatalysts combined with BiOX.

In this work, p-n heterojunction photocatalysts were synthesized by grinding SnO_2_ and BiOI mixtures at different ratios (denoted as BiOI/SnO_2_) for NO removal. The facile synthesis method enabled the environmental application of photocatalysts. The photocatalytic performance of the BiOI/SnO_2_ catalysts was evaluated. The structure, morphology, chemical state, and optical properties of BiOI/SnO_2_ were characterized. In addition, the photocatalytic NO degradation mechanism was illustrated based on in situ diffuse reflectance infrared Fourier transform spectroscopy (DRIFTS) analysis. This work provides a strategy for exploiting highly efficient visible-light-driven heterojunction photocatalysts to control air pollution.

## 2. Materials and Methods

### 2.1. Materials

Tin chloride pentahydrate (SnCl_4_·5H_2_O) was purchased from Sinopharm Chemical Reagent Co., Ltd. (Shanghai, China). Sodium hydroxide (NaOH) and potassium iodide (KI) were obtained from Chongqing Chuandong Chemical Co., Ltd. (Chongqing, China). Bismuth nitrate pentahydrate (Bi(NO_3_)_3_·5H_2_O) was purchased from Aladdin Biochemical Technology Co., Ltd. (Shanghai, China). Ethanol was purchased from Chengdu Chron Chemicals Co., Ltd. (Chengdu, China). NO (100 ppm) and dry air consisting of N_2_ and O_2_ with a 4:1 volume ratio were supplied by Chongqing Rising Gas (Chongqing, China). All the chemicals were of analytical grade and were used without further purification.

### 2.2. Synthesis of p-BiOI/n-SnO_2_ Heterojunction

**Figure 1:**
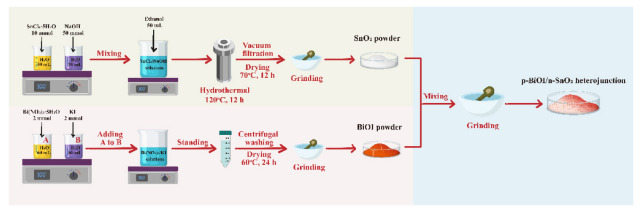
Fabrication procedure of p-BiOI/n-SnO_2_ heterojunction.

The synthesis procedure for preparing the p-BiOI/n-SnO_2_ heterojunction is displayed in Figure 1. SnO_2_ was prepared by hydrothermal method. Briefly, 10 mmol SnCl_4_·5H_2_O and 5 mmol NaOH were separately dissolved in 50 mL deionized water and stirred for 30 min. Next, these two solutions were mixed together. The resulting mixture was stirred for another 30 min, and 50 mL of ethanol was then added to the mixed solution under stirring. This solution was transferred into a 200 mL stainless steel Telfon-lined autoclave, which was sealed, heated to 120 °C, and held at this temperature for 12 h. After cooling down to room temperature, the precipitates were collected by vacuum filtration and washed with deionized water and absolute ethanol. The obtained samples were dried at 70 °C for 12 h and then ground into SnO_2_ powder for further usage. BiOI was obtained through a facile precipitation method. First, 2 mmol Bi(NO_3_)_3_·5H_2_O and 2 mmol KI were separately dissolved in 60 mL deionized water, denoted as A solution and B solution, respectively. After dissolution was complete, solution A was added dropwise into solution B. The resulting mixture was stirred for 30 min. Precipitates were collected after 2 h of precipitation, and the precipitates were then washed three times with deionized water and absolute ethanol. The samples were dried at 60 °C for 24 h and then ground into SnO_2_ powder for further usage. A series of p-BiOI/n-SnO_2_ heterojunctions were synthesized by varying the BiOI mass to 15%, 30%, and 75% of the SnO_2_ mass, and these samples were denoted as *X*%B-S (*X* = 15, 30, 75). For comparison, pristine SnO_2_ and BiOI were also prepared.

### 2.3. Characterization

X-ray diffraction (XRD) patterns of the samples were measured to characterize their structure and crystallinity using a Rigaku Miniflex II diffractometer (Tokyo, Japan) with a Cu Kα radiation source (λ = 1.5406 Å) and 2θ scanning speed of 10° min^−1^. The elemental composition and corresponding valence states on the sample surfaces were determined by X-ray photoelectron spectroscopy (XPS) using a Kratos Axis Ultra DLD spectrometer (Manchester, UK). Field emission scanning electron microscopy (SEM, JEOL model JSM-6335 F, Tokyo, Japan) was used to analyze the morphology of the samples. UV-vis diffuse reflectance spectra (UV-vis DRS) was performed with a Shimadzu UV-1800 spectrometer (Kyoto, Japan), and photoluminescence spectra (PL) were obtained using a JASCO Spectrofluorometer FP-8200 (Tokyo, Japan) to characterize the optical properties of the samples. Electron spin resonance (ESR) signals were recorded by a JESFA200 spectrometer (Tokyo, Japan), and 5,5-Mimethyl-1-pyrroline N-oxide (DMPO), 2,2,6,6-tetramethylpiperidine-1-oxyl (TEMPO), and 4-oxo-2,2,6,6-tetramethylpiperidine (4-oxo-TEMP) served as spin-trap reagents to trap photogenerated hydroxyl radicals (·OH) as well as superoxide anion radicals (·O_2_^−^), electrons, and singlet oxygen (^1^O_2_), respectively.

### 2.4. Photocatalytic NO Oxidation

The photocatalytic activities of the synthesized catalysts for NO oxidation were evaluated by measuring their gas-phase NO removal efficiency in a continuous flow reactor with dimensions of 30 cm × 15 cm × 10 cm. In detail, 0.1 g of the catalyst was ultrasonically dispersed and uniformly coated onto two glass culture dishes with diameters of 12 cm, followed by vacuum drying at 60 °C for 30 min. NO from a compressed gas cylinder (15 mL min^−1^) was diluted to a concentration of 500 ppb by an air stream (2.4 L min^−1^), which was continuously bubbled into the reactor. After adsorption–desorption equilibrium was achieved, a 150 W commercial tungsten halogen lamp (UV cutoff filter, λ ≥ 420 nm) was turned on to initiate the photocatalytic NO oxidation. The concentrations of NO and NO_2_ were measured by a NO*_x_* analyzer (Thermo Scientific, 42i-TL, Waltham, MA, USA) and sampled every minute for 30 min. The removal efficiency (η) of NO was calculated with the equation ${\eta =(1-C/C}_{0})\times 100\%$, where $C_{0}$ and C represent the concentrations of NO in the inlet and outlet, respectively. In addition, the catalyst stability was evaluated by performing NO oxidation five consecutive times following the same procedure. The photocatalyst did not undergo any treatment at the end of each cycle, and photocatalytic oxidation was initiated by visible light irradiation after adsorption–desorption equilibrium was achieved under dark conditions.

### 2.5. In Situ DRIFTS Analysis

In situ diffuse reflectance Fourier transform infrared spectroscopy (DRIFTS) measurements were performed in an in situ diffuse-reflectance cell (Harrick, New York, NY, USA) in a TENSOR II FT-IR spectrometer (Bruker, Ettlingen, Germany) to investigate the adsorption and reaction behavior of NO over the catalysts. Before measurements were performed, the obtained samples were pretreated for 20 min at 110 °C in a high-temperature reaction chamber to remove water, carbon dioxide, and carbohydrates from the catalyst surface. The baseline was recorded before injecting the reaction gas into the reaction chamber. The flow rate of the gas mixture (He, O_2_, and NO) was 100 mL min^−1^, and the concentration of NO was 50 ppm diluted by O_2_. After adsorption equilibrium was achieved, each catalyst was illuminated by visible light to initiate the photocatalytic reaction. Data were collected every 2 min for 30 min.

## 3. Results and Discussion

### 3.1. Photocatalytic Activity

**Figure 2:**
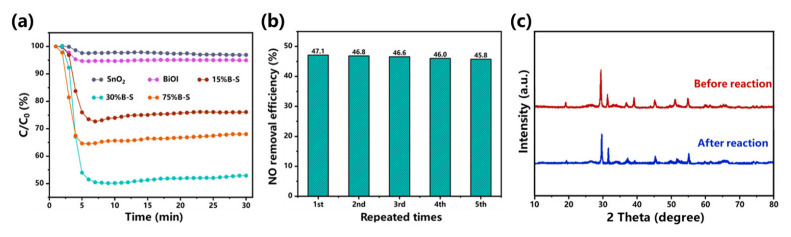
(**a**) Photocatalytic activity of as-synthesized catalysts for NO removal, (**b**) stability test of 30%B-S, and (**c**) XRD patterns of 30%B-S before and after cyclic test.

The photocatalytic performance of the prepared pristine BiOI, SnO_2_, and BiOI/SnO_2_ composites was evaluated under visible-light irradiation. Figure 2a shows the variation in NO concentration (expressed as a normalized ${C/C}_{0}$ concentration) in the presence of the prepared photocatalysts. The pristine uncombined SnO_2_ and BiOI exhibited weak photocatalytic activity, only oxidizing 3.1% and 5.1% of the NO, respectively. X%B-S exhibited enhanced photoactivity compared to pristine SnO_2_ and BiOI, indicating that heterojunction formation was beneficial for improving the photocatalytic activity [25]. The NO removal efficiency of the BiOI/SnO_2_ composites dramatically increased in the first 6 min and reached a maximum at 6–8 min. Remarkably, the visible-light driven-driven oxidation performance of X%B-S appeared to slightly decrease after 6–8 min, possibly due to the accumulation of reaction intermediates. The generation of intermediates could block the adsorption and active sites [26]. Optimal results were obtained using 30%B-S as the photocatalyst to achieve NO removal efficiency of up to 47.1%. The removal efficiency of 30%B-S was 96.3% and 47.2% higher than that of 15%B-S and 75%B-S, respectively. This was also better than the performance of other reported p-n heterojunction photocatalysts shown in Table 1, such as 20%BiOI/ZnWO_4_ (32.32%) [27], Bi_2_O_3_/Bi_2_O_2_CO_3_ (35%) [28], Bi_2_O_2_CO_3_/ZnFe_2_O_4_ (35%) [16], and 4%β-Bi_2_O_3_/CeO_2-δ_ (42.9%) [29]. In addition, the NO removal efficiency of 30%B-S did not significantly change after five consecutive cycles (Figure 2b), indicating the excellent stability and repeatability of its photocatalytic performance. The decrease in photocatalytic activity of 30%B-S (2.3%) was much lower than that of B-S-4 (15.5%) after four cycles [30]. XRD pattern further demonstrated that no obvious change occurred in the structure of 30%B-S during the cyclic test (Figure 2c). Therefore, the synthesized 30%B-S was an efficient and stable photocatalyst for long-term NO purification.

### 3.2. Microstructure and Chemical Composition

**Figure 3:**
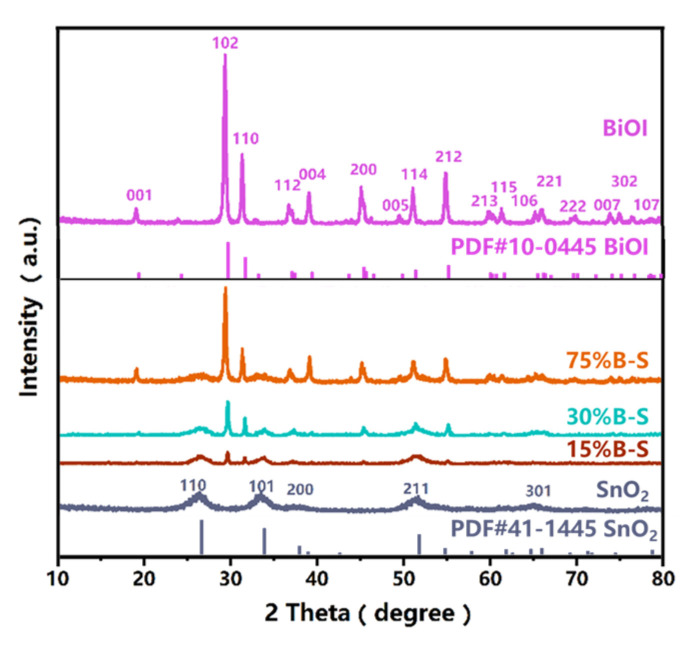
XRD patterns of BiOI, SnO_2_, and *X*%B-S (where *X* = 15, 30, and 75).

The crystallinity of pristine BiOI, SnO_2_, and the BiOI/SnO_2_ composites was identified by X-ray diffraction (XRD). As shown in Figure 3, all the characteristic diffraction peaks of SnO_2_ and BiOI were in good agreement with those pertaining to the SnO_2_ tetragonal phase (PDF#41-1445) and BiOI tetragonal phase (PDF#10-0445), respectively. The composites possessed all the characteristic peaks of pristine BiOI and SnO_2_ without any peak shift, implying that the crystal structures of the constituent monomers were not affected by the preparation of *X*%B-S. Moreover, with increasing BiOI content in *X*%B-S, the characteristic peaks pertained to BiOI became sharper and similar to those of pure BiOI, while the diffraction peaks corresponding to the (110) crystal plane of SnO_2_ gradually weakened. This indicated that the composite contained both BiOI and SnO_2_.

**Figure 4:**
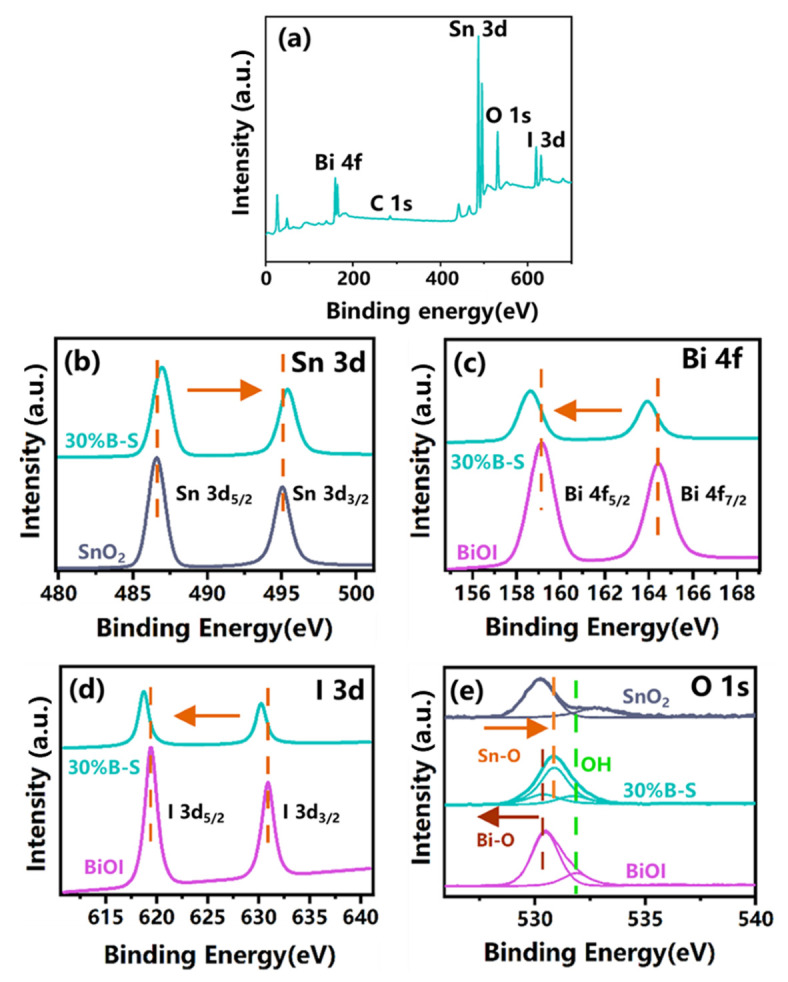
XPS survey spectrum of (**a**) 30%B-S. High-resolution XPS spectra of (**b**) Sn 3d, (**c**) Bi 4f, (**d**) I 3d, and (**e**) O 1s for BiOI, SnO_2_, and 30%B-S.

X-ray photoelectron spectroscopy (XPS) measurements were performed to further characterize the surface elemental compositions of pristine BiOI, SnO_2_, and 30%B-S. Figure 4a demonstrates the coexistence of Bi, Sn, O, and I elements in 30%B-S. The high-resolution Sn 3d spectra of SnO_2_ and 30%B-S (Figure 4b) depict one pair of strong peaks located at 486.44 and 494.96 eV. These peaks were attributed to Sn 3d_3/2_ and Sn 3d_5/2_, respectively, suggesting that the valence state of Sn was positive tetravalent [31]. Figure 4c shows that the Bi 4f spectra had two peaks at 159.02 and 164.32 eV, which were associated with Bi 4f_7/2_ and Bi 4f_5/2_, respectively. These peaks were ascribed to the Bi^3+^ in neat BiOI [25]. In addition, the Bi^3+^ peaks slightly shifted to lower binding energies in the 30%B-S spectrum, which was possibly attributed to the electron transfer between BiOI and SnO_2_ [32]. A similar shift in binding energy was also observed in the I 3d spectra. As shown in Figure 4d, the I 3d peaks of BiOI at 618.94 and 630.42 eV were assigned to I 3d_5/2_ and I 3d_3/2_, respectively [33]. The O 1s spectra were resolved as three peaks at 530.42, 530.22, and 532.04 eV, which corresponded to the Sn-O bonds of SnO_2_ (530.38 eV) [34], Bi-O bonds of BiOI (530.31 eV) [25], and H-O bonds [35], respectively (Figure 4e). In general, the binding energies of the Bi 4f, O 1s, and I 3d peaks of 30%B-S shifted to lower values in comparison with neat BiOI. Meanwhile, the binding energies of the Sn 3d and O 1s peaks of 30%B-S shifted to higher values compared to those in neat SnO_2_. This demonstrated the existence of electron transfer from BiOI to SnO_2_ and the formation of a p-n heterojunction [36].

**Figure 5:**
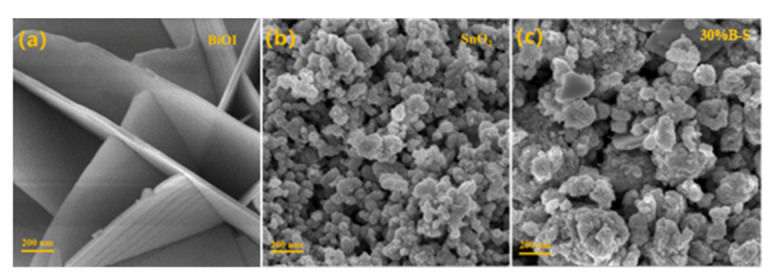
SEM images of (**a**) BiOI, (**b**) SnO_2_, and (**c**) 30%B-S.

The morphological features of pristine BiOI, SnO_2_, and 30%B-S were observed by scanning electron microscopy (SEM). Figure 5a shows that BiOI possessed thin regular nanosheets (thickness: 50–60 nm) with smooth surfaces, indicating its good crystallinity and uniformity. Figure 5b shows that SnO_2_ consisted of nanoparticle aggregates, with average nanoparticle sizes ranging from 20 to 30 nm. The BiOI/SnO_2_ composite shown in Figure 5c retained the same nanoparticle aggregate morphology.

### 3.3. Optical and Photoelectrochemical Properties

**Figure 6:**
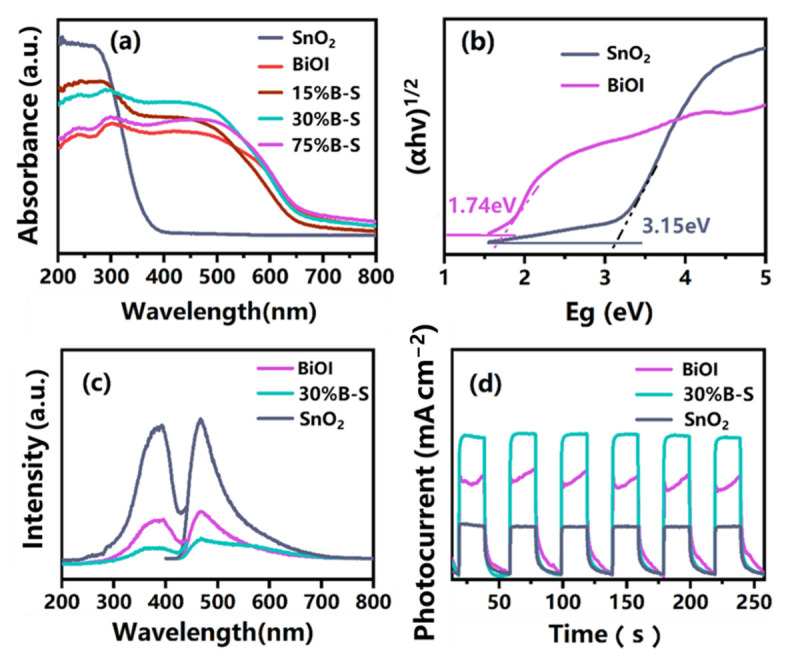
(**a**) UV-vis diffuse reflectance spectra (UV-vis DRS) of prepared samples, (**b**) plot of ${\left(\alpha hv\right)}^{1/2}$ versus light energy (hv) for BiOI and SnO_2_, (**c**) photoluminescence spectra (PL), and (**d**) photocurrent density of BiOI, SnO_2_, and 30%B-S.

According to UV-vis diffuse reflectance spectra (UV-vis DRS) analysis (Figure 6a), the pristine SnO_2_ only responded to ultraviolet light, with its absorption edge located at around 380 nm. However, the pristine BiOI responded in both the ultraviolet and visible light regions, with an absorption edge of 720 nm. Compared with neat SnO_2_, the optical absorption edge of *X*%B-S (*X* = 15, 30, 75) exhibited a redshift. Moreover, *X*%B-S (especially 30%B-S) possessed the better light harvesting performance compared with pristine BiOI and SnO_2_. These results can be ascribed to the formation of BiOI-SnO_2_ heterojunctions [37]. The optical band edges of BiOI and SnO_2_ were calculated using the Tauc equation ${\alpha hv=A(hv-E}_{g}{)}^{n/2}$, where α, h, v, and $E_{g}$ are the absorption coefficient, Planck’s constant, light frequency, and band gap energy, respectively. A is a constant, while n is defined by the optical transition of the semiconductor (namely, n=1 for direct transition and n=4 for indirect transition) [38]. Typically, indirect transition occurs in BiOI and SnO_2_, so n should equal 4 [39,40]. The band gap energy can thus be estimated by the intercept of the tangents in a plot of ${\left(\alpha hv\right)}^{1/2}$ versus light energy (hv), as shown in Figure 6b. The band gap energies of BiOI and SnO_2_ were 1.74 and 3.15 eV, respectively. The band edge energies of the conduction band (CB) and valence band (VB) of BiOI and SnO_2_ were further estimated by the following empirical formulas:

(1) $$E_{VB}{=X-E}^{e}+0{.5E}_{g}$$

(2) $$E_{CB}{=E}_{VB}{-E}_{g}$$

where $E^{e}$ represents the energy of free electrons on the hydrogen scale (~4.5 eV), $E_{g}$ is the semiconductor band gap, and X is the absolute electronegativity of the semiconductor, expressed as the geometric mean of the electronegativity of the constituent atoms. The values of X for BiOI and SnO_2_ were calculated to be 5.99 eV and 6.25 eV [40,41], respectively. Hence, the $E_{VB}$ and $E_{CB}$ of BiOI were calculated to be ca. 2.36 eV and 0.62 eV, while those of SnO_2_ were about 3.325 eV and 0.175 eV, respectively.

Figure 6c shows the photoluminescence spectra (PL) curves of the prepared samples, which were used to evaluate the separation and recombination efficiencies of photoexcited electron-hole pairs. The peak intensities of 30%B-S were significantly lower than those of neat BiOI and SnO_2_, demonstrating that BiOI doping significantly inhibited the recombination of photogenerated charge carriers. Photocurrent response tests revealed the relatively low photocurrent response of individual BiOI and SnO_2_ (Figure 6d). The photocurrent density of 30%B-S was enhanced by 1.5 and 0.5 times compared to neat SnO_2_ and BiOI, respectively, suggesting the effective separation efficiency of electron-hole pairs in the BiOI-SnO_2_ heterojunction [42]. This was in accordance with PL analysis.

### 3.4. Enhanced Photocatalytic Oxidation Mechanism

**Figure 7:**
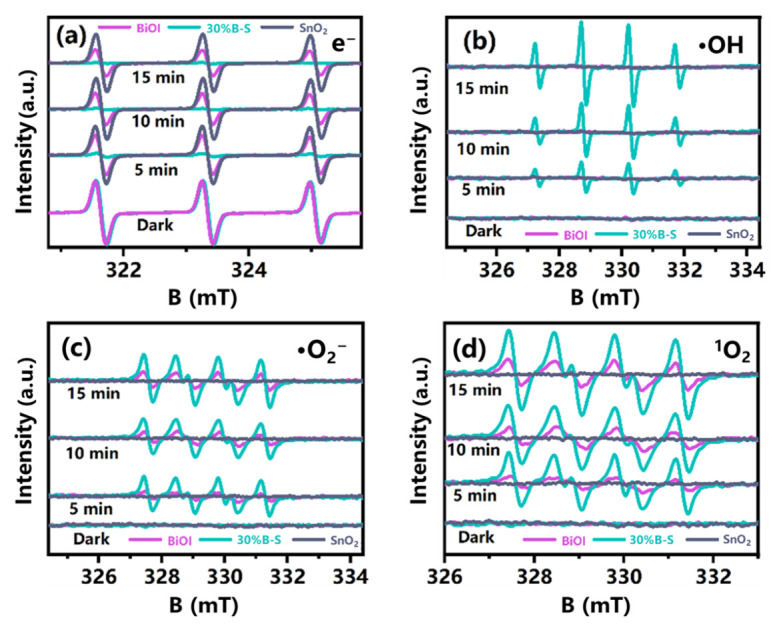
Spin-trapping electron spin resonance (ESR) spectra of (**a**) e^−^, (**b**) ·OH, (**c**) ·O_2_^−^, and (**d**) ^1^O_2_ for the BiOI, SnO_2_, and 30%B-S samples in the dark and under visible light illumination.

ESR analysis was used to detect the generation of active species with strong oxidant capacity, including e^−^, ·OH, ·O_2_^−^, and ^1^O_2_ on the catalyst surfaces [43]. Unlike neat BiOI and SnO_2_, the trapped e^−^ signal of 30%B-S sharply declined after illumination (Figure 7a). This was potentially because the electrons were consumed to generate the reactive oxygen species [44]. As illustrated in Figure 7b–d, the ESR signals of the radicals were not detected after illuminating neat SnO_2_ with visible light. This was because the large band gap of neat SnO_2_ could not be adequately excited to generate charge carriers. The ·OH, ^1^O_2_, and ·O_2_^−^ signal densities of 30%B-S were significantly stronger than those of pristine BiOI. In the dark, almost no distinct characteristic peaks were observed, suggesting that few or no ·OH, ^1^O_2_, and ·O_2_^−^ species were produced. Notably, after illumination for 2 min, four similar characteristic peaks appeared in the 5,5-dimethyl-1-pyrroline N-oxide (DMPO)-·OH spectrum of 30%B-S, with a peak intensity ratio of 1:2:2:1 (Figure 7b). The DMPO-·OH signals increased with increasing visible light illumination time, indicating the rapid generation of ·OH during the photocatalytic process. This was possibly because the BiOI doped in SnO_2_ promoted the activation of H_2_O molecules and the separation of spatial charges, leading to the transformation of H_2_O and the generation of ·OH species. Typical peaks of the DMPO superoxide adduct, such as DMPO-·O_2_^−^, and the 4-oxo-2,2,6,6-tetramethylpiperidine (4-oxo-TEMP) singlet oxygen adduct, such as 4-oxo-TEMP-^1^O_2_, were detected in both neat BiOI and 30%B-S. A strong response was achieved by 30%B-S (Figure 7c,d). Thus, the production of ·O_2_^−^ and ^1^O_2_ was significantly enhanced by BiOI doping, indicating the important roles of ·O_2_^−^ and ^1^O_2_ in the photocatalytic oxidation of NO.

**Figure 8:**
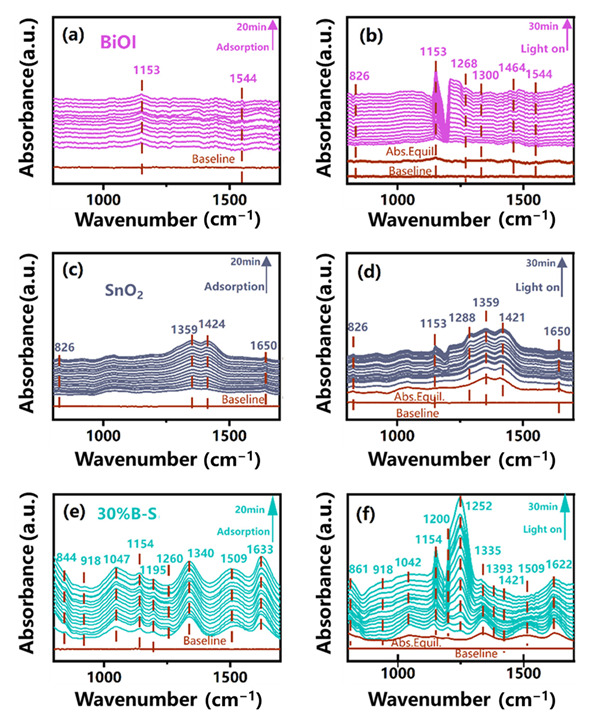
In situ DRIFTS spectra of NO adsorption and visible-light reaction processes over (**a**,**b**) BiOI, (**c**,**d**) SnO_2_, and (**e**,**f**) 30%B-S.

In situ DRIFTS was used to explore the NO photocatalytic oxidation pathway of the BiOI-SnO_2_ heterojunction. Figure 8a,c,e show the adsorption bands of species related to NO under dark ambient conditions for BiOI, SnO_2_, and 30%B-S, respectively. NO can interact with the nitrogen or oxygen atoms on the SnO_2_ surface to produce cationic dimer, two forms of nitrosyls bound to surface tin atoms and a nitrite Sn-O-NO-species [45]. The layered structure of BiOI nanoplate can provide the sufficient surface area for NO adsorption. The combination of SnO_2_ and BiOI contributed to the formation of more reactive sites for the NO adsorption [46], leading to the generation of more species on 30%B-S compared with BiOI and SnO_2_.The peaks located at 1633 cm^−1^ and 844 cm^−1^ in Figure 8e were respectively ascribed to the adsorbed NO and NO_2_^−^ generated on the surface of 30%B-S [47,48]. Moreover, the typical absorption peak of NO_3_^−^ (1340 cm^−1^) was detected in 30%B-S [8], which was attributed to the oxidation of NO by the active radicals adsorbed on the surface of the BiOI-SnO_2_ heterojunction. The NO_3_^−^ peak remained stable over the adsorption period, indicating that the oxidation reaction only occurred at the moment NO was absorbed. Then adsorption equilibrium was reached. The adsorbed NO preferentially attacked surface ·OH groups, generating NO^−^ and NOH through a reaction (i.e., 3NO + OH^−^ = NO_2_ + NO^−^ + NOH). The N-O-H was detected at 1154 cm^−1^ [49], similar to the absorption band of NO_3_^−^. After achieving adsorption–desorption equilibrium and under light illumination, bands at 1544 cm^−1^ and 1359 cm^−1^ corresponding to NO_2_ were observed in the BiOI and SnO_2_ spectra [50,51], respectively, while the NO_3_^−^ signal was undetectable (Figure 8b,d). More reactions were excited on the surface of 30%B-S under light irradiation, as indicated by appearance of increased absorption peaks (Figure 8f). This suggested that the formation of the heterojunction strengthened the visible light absorption capacity of the photocatalyst. The distinct absorption bands of NO_2_^−^ and NO_3_^−^ (861 cm^−1^ and 1252 cm^−1^) were observed [8,52], and the signal response of NO_3_^−^ rapidly increased with increasing light irradiation time. No NO_2_ peak was detected, revealing that 30%B-S heterojunction can efficiently transform NO into harmless NO_3_^−^ products.

**Figure 9:**
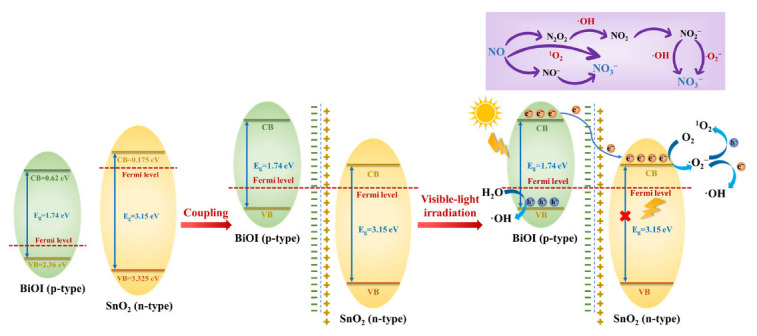
The proposed photocatalytic mechanism of NO oxidation by the BiOI/SnO_2_ heterojunction.

Considering these experimental results and theoretical analysis, a possible improved photocatalytic mechanism of NO oxidation over the p-BiOI/n-SnO_2_ heterojunction was proposed, as depicted in Figure 9. BiOI is a p-type semiconductor with a Fermi level (E_f_) similar to the VB, and SnO_2_ is a typical n-type with the E_f_ located near the CB. The coupling of BiOI with SnO_2_ enables the energy bands of BiOI to increase, while the energy bands of SnO_2_ decrease. Once the E_f_ of BiOI and SnO_2_ shift to the same level and reach equilibrium, a p-n heterojunction is formed. Ultimately, the CB of BiOI shifts to an energy level higher than that of SnO_2_, leading to the migration of photoexcited electrons from the CB of BiOI to SnO_2_. The driving force of this migration is the energy difference between the CB of BiOI and SnO_2_ under visible light irradiation. The electrons gathered on the CB of SnO_2_ convert O_2_ into ·O_2_^−^ and ·OH. ^1^O_2_ is generated by the reaction of ·O_2_^−^ with photogenerated holes. Moreover, the residual holes in the VB of BiOI oxidize H_2_O to produce ·OH. The generation of an internal electric field and construction of the p-n heterojunction changes the transmission pathway of the photo-induced charge carriers and strengthens the separation of electron-hole pairs, significantly promoting visible light photocatalytic NO oxidation.

## 4. Conclusions

A p-BiOI/n-SnO_2_ heterojunction with excellent activity, stability, and selectivity was successfully synthesized in this work. Among the prepared heterojunctions, the 30%B-S photocatalyst exhibited the most enhanced photoreactivity under visible light illumination, with a NO removal efficiency that was 96.3% and 47.2% greater than that of 15%B-S and 75%B-S, respectively. In addition, the NO removal efficiency of 30%B-S did not noticeably change after five cycles. The experimental results and theoretical analysis confirmed that NO was transformed into harmless NO_3_^−^ as the main final product over the p-BiOI/n-SnO_2_ heterojunction. The heterojunction changed the transmission pathway of photogenerated carriers and promoted the separation of electron-hole pairs, resulting in remarkable photocatalytic performance for NO purification. Overall, the proposed p-BiOI/n-SnO_2_ heterojunction photocatalyst shows good promise for the efficient oxidation of NO to NO_3_^−^.

## Figures and Tables

**Table 1:** NO removal performance of reported p-n heterojunction photocatalysts.

Photocatalyst	Dosage (mg)	Light Type	NO Concentration (ppb)	Time (min)	Removal Ratio (%)	Ref.
20%BiOI/ZnWO_4_	100	300 W xenon lamp (λ ≥ 420 nm)	430	30	32.32	[27]
Bi_2_O_3_/Bi_2_O_2_CO_3_	200	300 W xenon lamp (λ ≥ 420 nm)	400	30	35	[28]
Bi_2_O_2_CO_3_/ZnFe_2_O_4_	−	300 W xenon lamp (λ ≥ 420 nm)	400	30	35	[16]
4%β-Bi_2_O_3_/CeO_2-δ_	100	300 W xenon lamp (λ ≥ 420 nm)	430	30	42.9	[29]
30%B-S	100	150 W tungsten halogen lamp (λ ≥ 420 nm)	500	30	47.1	This work

## Data Availability

The data presented in this study are available on request from the corresponding author. The data are not publicly available due to privacy.
